# Organizing

**Published:** 1897-12

**Authors:** 


ORGANIZING.—It is very gratifying to note that at the meeting of the Brazos Valley Medical Society, held at Navasota on the 10th of November (ult.), twenty new members were added to the roll. The good work goes bravely on. Let other societies now come up with as good a showing. It is gratifying also to note that the Brazos Valley Medical Society in pursuance of the TEXAS MEDICAL JOURNAL'S advice, has passed strong resolutions condemning the physicians' occupation tax. Let every society adopt such resolutions, and let's try to get the law repealed.

Drs. M. M. Smith and A. N. Denton, of this city, have each purchased an interest in the *Texas Medical News*, formerly *Texas Sanitarian*, and each made his bow in the November issue, a very creditable edition indeed. If there is "wisdom in a multitude of counsel" there must be wisdom in a multitude of editors, and we look for a red-hot competitor.

---

The Texas Medical Journal has received several letters commendatory of its article defending Dr. Guiteras, and especially our remarks anent the multitude of indiginous yellow fever experts. If this scare has done no good in other directions it has revealed to us a wealth of resources heretofore unexpected. We did not know Texas was so well off. Why the woods are full of 'em, and should Texas be subject to another scare—imaginary or real—all alien experts are now put on notice that they are not wanted. The home article is on hand in supply more than equal to any demand.

